# Women in Selected Communities of Punjab, India Have a High Prevalence of Iron, Zinc, Vitamin B12, and Folate Deficiencies: Implications for a Multiply-Fortified Salt Intervention

**DOI:** 10.3390/nu15133024

**Published:** 2023-07-03

**Authors:** Yvonne E. Goh, Mari S. Manger, Mona Duggal, Reena Das, Shipra Saklani, Surbhi Agarwal, Deepmala Budhija, Manu Jamwal, Bidhi L. Singh, Neha Dahiya, Hanqi Luo, Julie M. Long, Jamie Westcott, Nancy F. Krebs, Rosalind S. Gibson, Kenneth H. Brown, Christine M. McDonald

**Affiliations:** 1 Division of Gastroenterology, Hepatology and Nutrition, Department of Pediatrics, University of California, San Francisco, CA 94609, USA; yvonne.goh@ucsf.edu (Y.E.G.); mari.manger@izincg.org (M.S.M.); 2 International Zinc Nutrition Consultative Group, San Francisco, CA 94609, USA; nancy.krebs@cuanschutz.edu (N.F.K.); khbrown@ucdavis.edu (K.H.B.); 3 Post Graduate Institute of Medical Education & Research, Chandigarh 160012, India; monaduggal2@gmail.com (M.D.); reenadaspgi@hotmail.com (R.D.); sheipra@gmail.com (S.S.); surbhiagarwal171990@gmail.com (S.A.); deepmala_b@yahoo.com (D.B.); manujamwal25@gmail.com (M.J.); singhbidhilord1990@gmail.com (B.L.S.); drnehadahiya@gmail.com (N.D.); 4 Rollins School of Public Health, Emory University, Atlanta, GA 30322, USA; hanqi.luo@emory.edu; 5 Department of Pediatrics—Section of Nutrition, University of Colorado School of Medicine, Aurora, CO 80045, USA; julie.long@cuanschutz.edu (J.M.L.); jamie.westcott@cuanschutz.edu (J.W.); 6 Department of Human Nutrition, University of Otago, Dunedin 9054, New Zealand; rosalind.gibson@otago.ac.nz; 7 Department of Nutrition and Institute for Global Nutrition, University of California, Davis, CA 95616, USA

**Keywords:** multiply-fortified salt, micronutrient status, food fortification, dietary intake, undernutrition

## Abstract

Dietary intake and biomarkers of micronutrient status of 100 non-pregnant women of reproductive age (NPWRA) were assessed to determine optimal levels of iron, zinc, vitamin B12, and folic acid to include in multiply-fortified salt (MFS) that will be evaluated in an upcoming trial. Weighed food records were obtained from participants to measure intake of micronutrients and discretionary salt, and to assess adequacy using Indian Nutrient Reference Values (NRVs). Statistical modeling was used to determine optimal fortification levels to reduce inadequate micronutrient intake while limiting intake above the upper limit. Fasting blood samples were obtained to assess iron, zinc, vitamin B12, and folate status. In usual diets, inadequate intake of iron (46%), zinc (95%), vitamin B12 (83%), and folate (36%) was high. Mean intake of discretionary salt was 4.7 g/day. Prevalence estimates of anemia (37%), iron deficiency (67%), zinc deficiency (34%), vitamin B12 insufficiency (37%), and folate insufficiency (70%) were also high. Simulating the addition of optimized MFS to usual diets resulted in percentage point (pp) reductions in inadequate intake by 29 pp for iron, 76 pp for zinc, 81 pp for vitamin B12, and 36 pp for folate. MFS holds potential to reduce the burden of micronutrient deficiencies in this setting.

## 1. Introduction

The burden of iron, zinc, vitamin B12, and folate deficiencies among women of reproductive age (WRA) in India is high. Recently updated national estimates for India show that 37%, 31%, 29%, and 58%, of WRA are deficient in iron, zinc, vitamin B12 and folate, respectively [1]. However, these national averages mask striking geographical and socioeconomic disparities. A survey in Haryana showed that, among WRA, the prevalence of iron deficiency (87%), vitamin B12 deficiency (58%), and folate insufficiency (79%) was much higher than the national average [2,3]. In addition, India’s 2016–2018 Comprehensive National Nutrition Survey (CNNS) found that while 31% of adolescents were deficient in zinc nationwide; in Punjab, this prevalence was 52% [4]. Micronutrient deficiencies during the preconception and antenatal period increase the risk of adverse pregnancy and birth outcomes [5]. Anemia and inadequate iron status during pregnancy are associated with preterm birth, small-for-gestational age, low birth weight, poor cognitive development in children, and greater maternal and child mortality [6,7,8,9]. Zinc is essential for immune health, reproductive function, growth, and development, and its deficiency during pregnancy has been associated with an increased risk of preterm birth [10,11]. Vitamin B12 and folate are essential for DNA synthesis and neurological development, and deficiencies increase the risk of miscarriage and congenital abnormalities, including neural tube defects [12]. Evidence of the harmful effects of micronutrient deficiencies among WRA shows that interventions to reduce the burden of micronutrient deficiencies remain a public health priority in India.

Large-scale food fortification (LSFF) is an effective, low-cost, and safe strategy to address micronutrient deficiencies at the population level [13,14]. Among the food vehicle options available in resource-poor settings, salt is considered effective because it is relatively inexpensive, consumed in fairly consistent amounts across population sub-groups, and has well-established processing and distribution systems [13]. Universal salt iodization programs have been successful in many settings and helped to significantly reduce iodine deficiency [13]. In India, the coverage of iodized salt is more than 90%, making it a particularly attractive fortification vehicle [15]. In recent years, evidence of the impact of salt fortified with iodine and iron—often referred to as doubly-fortified salt (DFS)—on iron status has accumulated, with a recent review and meta-analysis showing reductions in the prevalence of anemia and iron deficiency anemia by 41% and 63%, respectively [16].

Novel technology now permits the fortification of salt with multiple micronutrients: iron, zinc, vitamin B12, and folic acid, in addition to iodine, referred to as multiply-fortified salt (MFS) [17]. The Multiply-Fortified Salt (MFS) study in Punjab, India, will evaluate the effect of MFS vs. standard iodized salt (IS) on the micronutrient status of non-pregnant women of reproductive age (NPWRA) and preschool-aged children (12–59 months) in Punjab, India in a randomized, controlled, community-based trial. In preparation for this trial, a formative cross-sectional study among NPWRA in Mohali district, Punjab, was conducted to assess: (1) the prevalence of inadequate micronutrient intake and micronutrient deficiencies, (2) average discretionary salt intake, and (3) the optimal levels of micronutrients to be added to the MFS. The current paper reports the results of these formative assessments, which informed the design of the MFS trial.

## 2. Materials and Methods

### 2.1. Study Design and Selection of Study Participants

The study was a cross-sectional study conducted in the district of Mohali, also known as the Sahibzada Ajit Singh Nagar, in the state of Punjab, India, between December 2020 and February 2021. This peri-urban district is located 21 km from Chandigarh, the state capital, and has an estimated population of 994,628 people distributed across 383 villages and eight municipalities [18]. The adult literacy rate in the district is high at 83%, with near-universal access to electricity and potable water [19].

**Figure 1:**
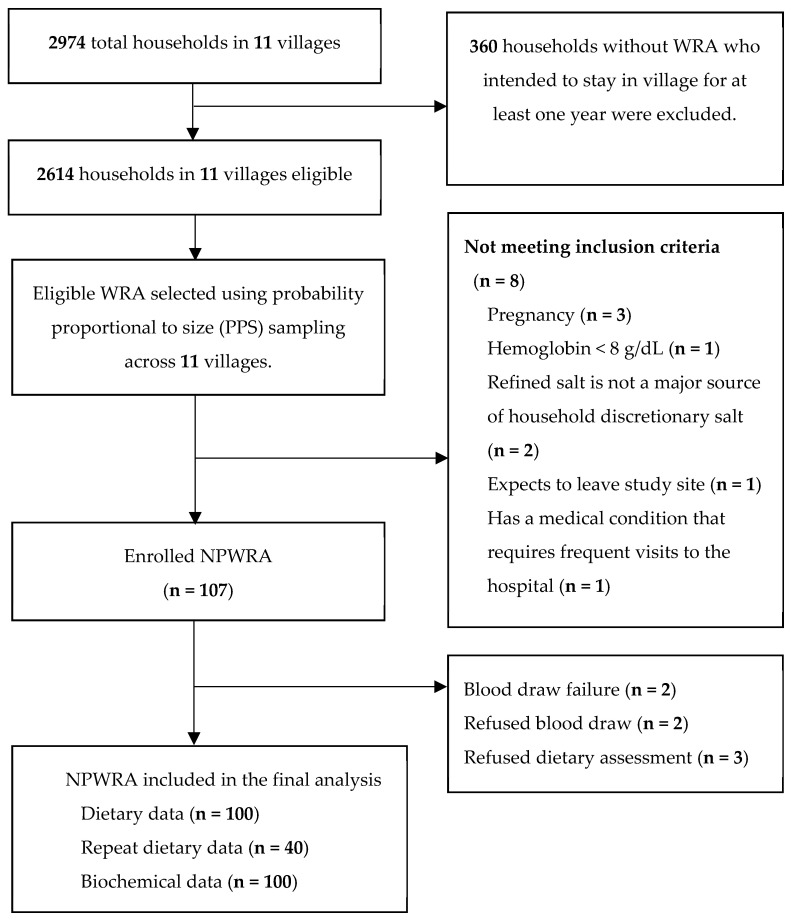
Flow of study participants.

Potentially eligible participants were NPWRA (18–49 years) living in the district. A census of all households (*n* = 2974) was carried out in a subset of 11 villages (Figure 1). Households that did not have a WRA and did not plan to stay in the area for at least one month were excluded from the sampling frame, reducing the number of potentially eligible households to 2614. Probability proportional to size sampling (PPS) was used to select potential participants from each village’s list of eligible households. The field research team held community sensitization meetings with community leaders and potentially eligible participants to explain the study’s objectives and provide information about the study procedures. At the end of the meetings, potential participants were provided with study information sheets and screening appointment cards. Participants were asked to discuss their potential participation with their family members before the study team’s arrival for screening the next day. On the screening day, potential study participants were excluded from the study if they (1) had experienced nausea or vomiting in the past three days (symptoms of early pregnancy), (2) had a hemoglobin concentration less than 8 g/dL as measured from a finger prick blood sample using the Hemocue^®^ Hb 301 system (Angelholm, Sweden), (3) planned to leave the study area for one month or more in the next 12 months, (4) had any medical condition that required regular visits to a health facility, or (5) did not use refined salt as the primary source of household discretionary salt. After screening, eligible NPWRA interested in participating in the study provided informed consent, including assent to undertake anthropometric assessments in their children under five years, and were enrolled in the study.

### 2.2. Data Collection Procedures

Women who met the eligibility criteria and consented to participate as well as have their children’s anthropometrics assessed were enrolled in the study and scheduled for anthropometry and blood sample collection at a central location in the village. Data were collected on household socio-demographic characteristics, salt procurement and utilization practices, and household food security after enrolment [20]. Dietary intake was assessed in the home of all participants.

#### 2.2.1. Dietary Assessment

One-day in-home weighed food records were collected from all 100 NPWRA participants and repeated on a non-consecutive day approximately one week later among a sub-sample of 40 NPWRA [21]. On the dietary assessment day, for each food or beverage item, including discretionary salt and water consumed by the participant, the time, place, amount consumed, and the amount left over were recorded by field research assistants on paper forms. All foods, condiments and beverages consumed were weighed to the nearest 0.1 g using dietary scales (Atom, Bangalore, India). The scales were calibrated with standard weights before each dietary assessment session. Detailed recipes of mixed dishes consumed by participants prepared during the dietary assessment period were collected in real-time. For any left-over mixed dishes prepared the previous day, the recipe was recalled or estimated using raw ingredients (including water) to represent the final dish. A list of fortified foods, beverages, and snacks and their micronutrient composition available in the study area was compiled before the study commenced. If a participant consumed a fortified food this was carefully noted during the dietary assessment. Detailed information was also recorded on any vitamin, mineral, or herbal supplement that was taken by the participant on the dietary assessment day. Additional information about participant illness and whether it affected appetite on the day of dietary assessment was also collected. The dietary assessment days were randomly distributed across all weekdays and weekends. Discretionary salt intake was estimated using the weighed food record data, whereby any discretionary salt consumed from recipes or added at the time of consumption was directly weighed with dietary scales with a precision of 0.1 g.

#### 2.2.2. Blood and Urine Specimen Collection and Anthropometry

Women were asked to appear for the biochemical and anthropometric assessment in a fasted state (no food or beverages apart from water consumed within the past 8 h) between 6.30 am to 10.30 am at a central location in the village. Whole blood (11 mL) was drawn from each participant by trained phlebotomists using the trace-element free Safety-Multifly^®^ 21G tube (80 mm) into three different S-Monovettes^®^ (Sarstedt, Numbrecht, Germany) containing ethylenediaminetetraacetic acid (EDTA), heparin, or no additive. The phlebotomists used trace-element free SHOWA^®^ (Grainger, Denver, CO, USA) gloves during the blood draw procedures. All tubes were placed in electronic portable coolers maintained at 4 °C and transported to the field laboratory for processing. Morning spot urine samples (50 mL) were collected from each participant woman at home and transported to the field laboratory for processing.

A team of two trained individuals completed the anthropometric assessments of participant women and their children under five years of age following standard procedures [22]. All anthropometry equipment was calibrated with standard weights or length rods each morning before the anthropometric sessions. The height of women and their children 24–59 months of age was measured to the nearest 0.1 cm using a stadiometer (SECA 213, GmbH & Co. KG., Hamburg, Germany). The length of infants under 24 months of age was measured to the nearest 0.1 cm using an infantometer (SECA 417). Digital balances (SECA 803 & 354) were used to measure the weight of all women and children to the nearest 0.1 kg and 0.01 kg, respectively. Mid-upper arm circumference (MUAC) was measured to the nearest 0.1 cm in all women and children 6–59 months of age using non-stretchable measuring tapes (UNICEF). Anthropometry was assessed twice for all participants. However, for women, if there was a difference of 0.2 kg or more in weight, a difference of more than 0.5 cm in height, or a difference of more than 0.5 cm in MUAC, a third measurement was required. For children, if there was a difference of more than 0.05 kg in weight, a difference of more than 0.5 cm in length or height, or a difference of more than 0.5 cm in MUAC, a third measurement was required.

##### Processing and Analysis of Biological Specimens

First, 100 µL of whole blood was aliquoted from the EDTA tube into a cryovial containing 1% ascorbic acid for later analysis of red blood cell (RBC) folate and 3 µL of whole blood was used to test malaria Pf/Pv antigen using a malaria rapid test kit (SD Biosensor Healthcare PVT. Ltd., Haryana, India). After this, the whole blood samples in EDTA tubes were analyzed for complete blood count (CBC) at the field laboratory using an automated hematology analyzer (Sysmex XP-300, Kobe, Japan). The remaining whole blood was then centrifuged at 2000× *g* for 10 min to separate the plasma, which was then aliquoted for subsequent homocysteine analysis. Heparinized blood samples were also centrifuged at 2000× *g* for 10 min for the analysis of plasma zinc concentrations. To prevent contamination, the plasma samples were aliquoted under a hood using zinc-free materials. The blood sample in the additive-free monovette was left undisturbed for 30 min to allow the blood to clot, after which it was centrifuged at 2000× *g* for 10 min. The serum was then aliquoted into separate cryovials for the analysis of holotranscobalamin (holoTC), methylmalonic acid (MMA), ferritin, soluble transferrin receptor (sTfR), alpha-1-acid glycoprotein (AGP), C-Reactive Protein (CRP), vitamin B12, folate, and thyroglobulin.

Plasma zinc analysis was carried out in the Pediatric Nutrition Laboratory, Department of Pediatrics, Section of Nutrition, University of Colorado School of Medicine. Plasma zinc concentrations were determined by Inductively Coupled Plasma Mass Spectrometry (ICP-MS, Agilent Technologies 7700×, Santa Clara, CA, USA) [23]. Then, 330 µL samples were diluted with 5 ppb yttrium (1:350) and directly aspirated into ICP-MS. SeroNorm™ Trace Elements Serum Level 1 (#SR201413, Accurate Chemical and Scientific Corporation, Westbury, NY, USA) was used as an internal control. Calibration standards were prepared with Supelco TraceCert™ ICP zinc standard solution and internal standard solution with TraceCert™ ICP yttrium standard solution (#75594 and #75592 respectively, Aldrich Chemical Company, Inc., Milwaukee, WI, USA). Plasma was diluted 300 times using ~3 ppb yttrium solution in 2% HNO_3_. A standard curve was established with three zinc standards: 0.70 µg/mL, 1.00 µg/mL, and 1.25 µg/mL, and the zinc concentration in the serum sample was measured against the standard curve. The relative standard deviation of counts and concentration of three replicate measurements of a single sample was less than 1.0%. Internal controls, an in-house plasma pool and SeroNorm™ Trace Elements serum level 1 (#SR201413, Accurate Chemical and Scientific Corporation, Westbury, NY, USA), were analyzed after every five serum samples, alternating between the two, within each analytical run. The within assay and between assay precision was 4% and 12.3%, respectively, for the internal controls. The limit of detection was 0.1 µg/dL.

The aliquoted serum was analyzed for the following biomarkers at the Post Graduate Institute of Medical Education and Research, Chandigarh, India (PGIMER): ferritin, sTfR, AGP, CRP, vitamin B12, and thyroglobulin). Urinary iodine, urinary creatinine, plasma homocysteine, RBC folate and serum folate were also analyzed at PGIMER, Chandigarh. Ferritin, vitamin B12, and thyroglobulin were assessed by electrochemiluminescence immunoassay on Cobas E411 Analyzer (Roche Diagnostics, Germany) and sTfR, CRP, and AGP were assessed by particle-enhanced immunoturbidimetric assay on Cobas 8000 Analyzer (Roche Diagnostics, Germany). Urinary iodine was measured using human urinary iodine enzyme-linked immunosorbent assay (ELISA) Kits. Urinary creatinine was assessed on Cobas 8000 Analyzer (Roche Diagnostics, Germany). RBC and serum folate was analyzed using the microbiologic assay with chloramphenicol-resistant strains of *Lactobacillus rhamnosus* culture and a 5-methyltetrahydrofolate calibrator [24]. Quality control pools for low, medium, and high whole blood pools were performed, and aliquots were stored at −80 °C for each assay. For each run, a standard growth curve of the organisms was carried out. Four replicates were run for each sample to ensure the generation of accurate results. The growth of the organisms was then read as turbidity after incubating at 37 °C for 40–42 h. Plasma homocysteine was analyzed using the fully automated ADVIA Centaur XP immunoassay system (Siemens Healthineers, Germany). Serum holoTC was analyzed at St John’s Research Institute, Bangalore, India by Axis-Shield HoloTC ELISA (Axis-Shield Diagnostics Ltd.). Serum MMA was also analyzed at St John’s Research Institute, Bangalore, India. Then, 200 µL of serum was deproteinized with ethanol, and derivatized and extracted in a single step by the addition of methylchloroformate and toluene. The N(S)-methoxycarbonyl ethyl ester derivatives were then analyzed by selected-ion monitoring (SIM) mode by Gas Chromatography Mass Spectrometry (GCMS-SQ, 5975, Agilent Technologies, Santa Clara, CA, USA) [25].

### 2.3. Definition of Biochemical Outcomes

Anemia was defined as hemoglobin concentration < 12.0 g/dL and iron deficiency was defined according to cut-offs <15 µg/L and >8.3 mg/L for inflammation-adjusted serum ferritin and sTfR, respectively [9,26,27]. A plasma zinc concentration < 70 µg/dL defined low fasting plasma zinc concentrations [28,29]. Folate insufficiency was defined as RBC folate concentration < 748 nmol/L [30]. Vitamin B12 deficiency and insufficiency were defined as serum vitamin B12 < 150 pmol/L and 150–221 pmol/L, respectively [30]. Plasma homocysteine concentrations > 13 μmol/L were defined as elevated [30]. HoloTC < 35 pmol/L and MMA levels > 271 nmol/L also indicated vitamin B12 deficiency [31]. A composite indicator of vitamin B12 status (cB12) was calculated using the equation, cB12 = log_10_[(holoTC × B_12_)/(MMA × Hcy)] − [3.79/(1 + [age/230]^2.6^). Elevated levels (cB12 > 1.5), adequate levels (−0.5 < cB12 < 1.5), low levels (−1.5 < cB12 < −0.5), and possibly deficient (cB12 < −2.5) were used to define values observed [31]. CRP and AGP concentrations ≥ 5 mg/L and ≥1 g/L, respectively, indicated inflammation. Urinary iodine concentration < 100 ug/L was defined as inadequate [32].

### 2.4. Data Analysis

#### 2.4.1. Dietary Data Analysis

A study-specific food composition table (FCT) was compiled primarily using the Indian FCT [33]. Nutrient values for commonly eaten simple recipes not included in the Indian FCT, such as plain chapatti and cooked rice, were obtained from the Bangladesh FCT [34]. Vitamin B12, iodine, and phytate values of foods were not available in the primary FCTs and were imputed from the USDA FCT, Norwegian FCT, and the FAO/INFOODS/IZiNCG Global Food Composition Database for phytate, respectively, accounting for the difference in water contents of foods between the different FCTs [35,36,37]. Most of the foods captured in the FCTs were raw and, therefore, appropriate retention factors of nutrients after different cooking methods were applied using the EUROFIR database [38]. For mixed dishes, the mixed recipe method of calculating energy and nutrient values recommended by FAO and EUROFIR was used [38]. The food intake data and the compiled study-specific FCT were then used to calculate participants’ energy and nutrient intake. If a participant consumed a fortified food or a vitamin, mineral, or herbal supplement the additional micronutrients consumed were accounted for when calculating the nutrient intake for the participant. The SIMPLE macro tool, based on the National Cancer Institute (NCI) method, was used to estimate the distribution of usual dietary intake of iron, zinc, vitamin B12, and folate and their corresponding prevalence of inadequate and excessive intake [39,40]. The calculations were done using the 2020 Indian Nutrient Reference Values (NRVs) [41]. The study population was lacto-vegetarian, consumed no meat, organ meat, fish, or eggs, and had a high intake of legumes; therefore, the iron absorption from the diet was estimated to be 8% according to the Indian NRVs [41,42]. Inadequate intake of zinc, vitamin B12, and folate were estimated using the Estimated Average Requirement (EAR) cut-point method [21,43]. Inadequate iron intake was calculated using the EAR cut-point method on log-transformed iron intake, and the log-transformed Indian EAR for WRA was applied. Absorbed zinc and vitamin B12 were estimated using the Miller and Doets equations, respectively, and both the absorbed and total values of zinc and vitamin B12 were used to estimate inadequate intake [44,45]. Dietary folate equivalents (DFE = food folate + 1.7 ∗ folic acid) were used to estimate the inadequate intake of folate. The total intake of iron, zinc, and synthetic folic acid above the tolerable upper intake level (UL) was used to calculate the respective excessive intake. Since vitamin B12 has no established UL, excessive intake was not estimated. The balanced repeated replication (BRR) technique was used to compute standard errors for the estimates of inadequate and excessive intake [39].

To demonstrate the potential effect of salt fortification on inadequate and excessive intake in the current sample, a two-step model simulation referred to as the “shrink then add” approach was used [40]. The first simulation step involved modeling the effect of the four nutrients consumed from the usual diet only. The second step modeled the effect of the four micronutrients from food sources plus the anticipated amount of each micronutrient that the MFS would contribute based on the estimated intake of discretionary salt obtained from the weighed food records. The prevalence estimates of inadequate and excessive intake were reported for each simulation to demonstrate how much the intake of the MFS will potentially affect the baseline prevalence estimates of inadequate intake.

The MFS premix was manufactured before the modeling activity was conducted. Therefore, it was only possible to adjust the micronutrient content of the MFS by varying the premix: salt blending ratio. Our approach was to ensure that the simulated prevalence of excessive intake did not exceed >5% for any micronutrient. Given the micronutrient content of the women’s usual diet, iron was the key micronutrient that drove these analyses. The levels of iron in the MFS premix: salt blending ratio were varied from the maximum possible level (2.5 mg/g of salt) and in decreasing amounts to determine the fortification level that ensured the prevalence of excessive iron intake did not exceed 5%. The fortification level for iron dictated the corresponding fortification levels of zinc, vitamin B12, and folic acid (Table 1).

#### 2.4.2. Biochemical and Anthropometric Data Analysis

Boxplots and histograms were generated to visually check the biochemical data for outliers. Outliers that were plausible after careful investigation were included in the analysis. The percentages of participants falling below the pre-defined biomarker cut-offs were calculated and reported. Serum ferritin and sTfR were adjusted for inflammation using BRINDA regression equations [26,27]. The relations between serum zinc concentrations and CRP and AGP as biomarkers of inflammation were examined. Because there was no significant association, we did not adjust serum zinc concentrations for CRP or AGP. Our approach is consistent with those reported by the BRINDA consortium for WRA [46]. SAS version 9.4 was used for data analysis. WHO Anthro Survey Analyzer was used to calculate height-for-age (HAZ), weight-for-height (WHZ), and weight-for-age (WAZ) Z scores for children under five years [47]. The mean and SD were reported for HAZ, WHZ, WAZ, and MUAC. The mean and SD were reported for the women’s weight, height, BMI, and MUAC. Women with BMI values < 18.5 kg/m^2^, 18.5–24.9 kg/m^2^, and ≥25.0 kg/m^2^ were classified as underweight, healthy, and overweight, respectively. Child MUAC measurements were categorized according to values < 115 mm (severely acutely malnourished), 115–124 mm (moderately acutely malnourished), and ≥125 mm (normal).

#### 2.4.3. Ethics

The study protocol was approved by the Institutional Review Board, University of California San Francisco, Institutional Ethics Committee, Post Graduate Institute of Medical Education and Research, and Health Ministry’s Screening Committee (HMSC) of India.

## 3. Results

The flow of participants through the study is shown in Figure 1. Dietary and biochemical data were available for 100 WRA. The characteristics of the study participants are presented in Table 2. On average, women were 35 years old, with the majority (98%) having at least a middle school education. Participants belonged to households with an average size of six persons and a monthly household income of INR 12,000 (USD 1~INR 75). All participants were classified as food secure. More than half (55%) of the women were either overweight or obese (BMI ≥ 25 kg/m^2^). Women who were overweight or obese had a higher prevalence of elevated CRP (33%) and AGP (33%) compared to women of normal BMI with CRP (9%) and AGP (11%). About 59% of the children of participant women under five years of age were male. The mean age, HAZ, WAZ, and WHZ of the children were 32.0 ± 14.6 months, −0.7 ± 1.0, −0.6 ± 1.0, and −0.3 ± 1.0, respectively.

The prevalence of inadequate and excessive dietary intake of iron, zinc, vitamin B12, and folic acid in the usual diets including intake of micronutrient supplements and the projected changes after the introduction of MFS, according to the Indian NRVs, are presented in Table 3. The mean intake of iron, zinc, vitamin B12, and folate from the usual diet was 18.8 ± 1.3 mg/d, 7.6 ± 0.2 mg/d, 1.30 ± 0.1 µg/d, and 200.7 ± 5.8 µg DFE/d, respectively. The mean intake levels of absorbable zinc and vitamin B12 after the Miller and Doets equations were applied were 1.39 ± 0.0 mg/d and 0.5 ± 0.0 µg/d, respectively. The estimate of usual discretionary salt intake, which we used in modeling the prevalence of inadequate and excessive dietary intake, was 4.7 ± 0.8 g/d [48]. The modeling results showed that, given the study population’s discretionary salt intake, the introduction of MFS containing the optimized ratio of pre-mix to salt is expected to reduce the prevalence of inadequate intake of iron from 46% to 17%, zinc from 95% to 18%, vitamin B12 from 83% to 0%, and folate from 36% to 0%, while ensuring that less than 5% of NPWRA will have excessive intake of iron, zinc, and folic acid. The energy and macronutrient intake of participants are presented in the Supplementary Materials.

The biochemical assessment revealed a high prevalence of micronutrient deficiencies (Table 4). A total of 37%, 67%, and 35% of women had anemia, iron deficiency, and iron deficiency and anemia, respectively. Thirty-four percent of women had hypozincemia, and the prevalence of vitamin B12 deficiency and insufficiency combined was 60%. The composite indicator of vitamin B12 (cB12) also showed that 63% of women had low and possibly deficient levels of vitamin B12. The majority (70%) of women were folate insufficient (RBC folate < 748 nmol/L). None of the participants had urinary iodine levels < 100 μg/L. The prevalence of inflammation, CRP ≥ 5 mg/L, and AGP ≥ 1 g/L was 22% and 23%, respectively. The mean ± SD or median (IQR) of the micronutrient biomarkers are presented in the Supplementary Materials.

## 4. Discussion

The current study confirms a high prevalence of inadequate micronutrient intake and micronutrient deficiency among NPWRA in the Mohali district, Punjab. These results highlight the urgent need for innovative, cost-effective strategies to improve micronutrient status among vulnerable populations in India. Large-scale food fortification is one such strategy and this current population is likely to benefit from MFS.

The dietary intake data revealed a high prevalence of inadequate intake of iron (46%), zinc (95%), folate (36%), and vitamin B12 (83%) among our study population, which aligns with the results of the biochemical assessment. Modeling the results of micronutrient intake from the usual diet plus the additional micronutrients provided by the MFS shows that MFS holds great potential to substantially reduce the prevalence of inadequate intake. Recently, concerns have been raised about the potential risk of excessive intake of some micronutrients, especially iron, in the Indian population due to the introduction of multiple iron-fortified food vehicles, including rice [49]. This concern was directly addressed by the current study in two main ways. First, our dietary assessment accounted for the intake of micronutrients from fortified foods and supplements in the baseline estimates of micronutrient intake. Second, we modeled the prevalence of inadequate and excessive intake before and after the introduction of MFS based on discretionary salt intake estimates from the study population [48]. By taking these steps, we were able to identify the optimal fortification levels that maximize reductions in the prevalence of inadequate intake while ensuring the prevalence of excessive intake of each micronutrient does not exceed 5% after the introduction of MFS, as specified by WHO guidelines [50].

National-level surveys and other smaller studies directly corroborate the estimates of micronutrient deficiency and insufficiency from biochemical assessments reported in the current study. The National Family Health Surveys and the CNNS have reported a high burden of anemia (57%), iron (87%) and zinc (31%) deficiencies and folate (79%), and vitamin B12 (58%) insufficiencies in India [2,3,4,20]. A recent systematic review and meta-analysis of the micronutrient deficiency burden in India showed that 54% of the population had iron deficiency, 37% had folate deficiency, and 53% had a vitamin B12 deficiency [51]. Our results are consistent with the prevailing estimates that indicate a high burden of micronutrient deficiencies among WRA in India.

Our study has several strengths which deserve comment. We used a representative sample of the study population by conducting a census of all 11 villages in our sampling frame and using PPS sampling to randomly select NPWRA. We also employed gold-standard and comprehensive methods for assessing dietary intake, discretionary salt intake, and micronutrient status. In addition, we used novel statistical modeling to simulate the changes in inadequate and excessive micronutrient intake, assuming different fortification levels of the multiply-fortified salt.

One potential weakness of the study is the fact that though the future MFS trial will primarily be targeted toward NPWRA, the MFS study salt will be available to the entire household, and we have not modeled the effect of the MFS on excessive micronutrient intake of other household members. Excessive intake of zinc, vitamin B12, and folate was negligible in the baseline usual diets of NPWRA and is not a source of concern. However, assuming that discretionary salt intake is proportional to energy intake, the MFS will provide less than 50% of the RDA for iron for all other population groups. At our proposed fortification levels, the risk of excessive iron intake appears to be low across all population groups. Another limitation is that we could not use the full probability method to estimate inadequate iron intake in our sample. This method is recommended for growing children and menstruating women whose iron requirements are skewed. The Indian NRVs do not include distribution tables of usual iron intake and requirements, which prevented us from applying this method.

## 5. Conclusions

This study represents comprehensive formative research, which was used to inform the formulation of MFS that will be evaluated in an upcoming randomized, controlled, community-based trial. It also confirms the study population of NPWRA in Mohali district, Punjab, is nutritionally vulnerable and likely to benefit from the MFS intervention.

## Figures and Tables

**Table 1:** Proposed amounts of micronutrients delivered by 4.7 g/d of multiply-fortified salt in relation to the Indian EAR, RDA, and UL.

Nutrient	Proposed Fortification Level (per Gram of Salt)	Expected Average Amount of Micronutrients Provided by MFS per Day	India Nutrient Reference Values			
			EAR	RDA	UL	% of RDA Met by MFS
Iron	1.3 mg	6.0 mg	15 mg/day ^1^	29 mg/day	45 mg/day	21
Zinc	1.4 mg	6.5 mg	11 mg/day ^2^	13.2 mg/day	40 mg/day	49
Vitamin B12	0.6 µg	2.8 µg	2 µg/day	2.2 µg/day	---	127
Folic acid	52 µg	244 µg	180 µg/day ^3^	220 µg/day ^4^	1000 µg/day	111
Iodine	30 µg	141 µg	95 µg/day	140 µg/day	1100 µg/day	100

EAR: estimated average requirement; RDA: recommended daily intake; UL: tolerable upper intake level. ^1^ The EAR was estimated based on iron bioavailability of 8%, accounting for the high phytic acid content of most diets in India. ^2^ The bioavailability of zinc in Indian diets was estimated to be 23% across all age and gender groups of the Indian population. ^3, 4^ The EAR and RDA refer to folate.

**Table 2:** Socio-demographic characteristics of study participants ^1^.

	Mean ± SD, Median, IQR; or *n* (%)
Woman Characteristics (*n* = 100)	
Age, years	34.5 ± 6.9
BMI, kg/m^2^	26.4 ± 5.5
Underweight < 18.5 kg/m^2^	7 (7.0)
Normal 18.5–24.9 kg/m^2^	38 (38.0)
Overweight 25.0–29.9 kg/m^2^	31 (31.0)
Obese > 30 kg/m^2^	24 (24.0)
Mid-upper arm circumference, cm	29.8 ± 4.4
Number of children under 5 years ^2^	1.9 ± 1.2
Education completed	
None	2 (2.0)
Middle/Secondary	72 (72.0)
Diploma/postgraduate	26 (26.0)
Primary Occupation	
Homemaker	79 (79.0)
Professional/Clerical	12 (12.0)
Shop owner/Supplier of goods	9 (9.0)
Religion	
Sikh	80 (80.0)
Hindu	18 (18.0)
Muslim	2 (2.0)
Marital status	
Married	87 (87.0)
Separated/never married	13 (13.0)
Household Characteristics	
Monthly income (INR)	12,000 (10,000, 25,000)
Ownership of ration card	67 (67.0)
Category of ration card	
Below poverty line	41 (61.2)
Above poverty line	21 (31.3)
Other priority households	5 (7.5)
Household head, female	17 (17.0)
Land ownership, %	38 (38.0)
Number of people living in household ^3^	5.8 ± 2.1
Child Characteristics (*n* = 34)	
Sex, male	20 (58.8)
Age, months	32.0 ± 14.6
Height-for-age z-score ^4^	−0.7 ± 1.0
Weight-for-age z-score	−0.6 ± 1.0
Weight-for-height z-score	−0.3 ± 1.0
Mid-upper arm circumference, cm	
11.5–12.5 cm	1 (2.9)
≥12.5 cm	33 (97.1)

^1^ *n* = 100 unless otherwise noted. ^2^ Number of women who had ever given birth and were eligible to answer, *n* = 89. ^3^ A household was defined as a group of people who are primarily dependent on an individual person, currently eat from the same pot, and live under the same roof or in the same compound. ^4^ The number of children whose anthropometric indices were calculated, *n* = 34.

**Table 3:** Prevalence of inadequate and excessive intake of micronutrients in the usual diet and projected intake after the introduction of MFS.

Nutrients ^1^	Mean Intake ± SE	EAR ^2^	Inadequate Intake (%)	Excess Intake (%)
**Iron**				
Iron intake from usual diet, mg/d	18.8 ± 1.3	15 ^3^	46.0 ± 5.1 ^4^	2.2 ± 1.8
Projected iron intake from usual diet and MFS, mg/day	25.2 ± 1.4	15 ^3^	16.9 ± 4.4	4.9 ± 3.0
**Zinc**				
Zinc intake from usual diet, mg/d	7.6 ± 0.2	11	94.5 ± 1.9	0.0 ± 0.0
Projected zinc intake from usual diet and MFS, mg/d	14.1 ± 0.4	11	18.3 ± 4.03	0.0 ± 0.0
Absorbed zinc from usual diet, mg/d ^5^	1.39 ± 0.0	3	99.9 ± 0.1	0.0 ± 0.0
Projected absorbed zinc intake from usual diet and MFS, mg/d	7.8 ± 0.3	3	0.3 ± 1.1	0.0 ± 0.0
**Vitamin B12**				
Vitamin B12 intake from usual diet, µg/day	1.3 ± 0.1	2	82.6 ± 3.5	-
Projected vitamin B12 intake from usual diet and MFS, µg/day	4.1 ± 0.2	2	2.0 ± 2.6	-
Absorbed B12 from usual diet, µg/day ^6^	0.5 ± 0.0	1	93.6 ± 2.6	-
Absorbed B12 from usual diet and MFS, µg/day	1.5 ± 0.1	1	11.7 ± 4.1	-
**Folate**				
Folate intake from usual diet, µg DFE/d ^7^	200.7 ± 5.8	180	35.7 ± 4.6	0.0 ± 0.0
Projected folate intake from usual and MFS, µg DFE/d	613.1 ± 20.1	180	0.01 ± 0.26	1.9 ± 1.9

Values are mean ± standard error; level of fortificants added to salt: **iron** = **1.3 mg/g** salt; **zinc** = **1.4 mg/g** of salt; **folic acid** = **52.4 μg/g** of salt; and **vitamin B12** = **0.6 μg/g** of salt. ^1^ Nutrient intake included the intake of micronutrient supplements (iron: *n* = 1; folic acid: *n* = 2) and was adjusted for usual intake using the NCI method with the Simple Macro tool (39,40). ^2^ EARs were taken from the 2020 Indian Nutrient Reference Values (41). ^3^ EAR is based on an estimated bioavailability of 8% in Indian diets (41). ^4^ The EAR cut-point method was used to estimate inadequate intake after log transforming iron intake and EAR. ^5^ In lieu of the EAR, the physiological requirements based on estimated total endogenous losses was used. Absorbed zinc was calculated using the Miller equation (45). ^6^ The EAR for absorbed vitamin B12 is assumed to be 50% of the EAR of dietary vitamin B-12. Absorbed vitamin B12 was estimated using the Doets equation (46). ^7^ The unit reported for folate is DFE ug/d which is equivalent to food folate + 1.7 ∗ folic acid. Excessive folate intake was estimated using intake of folic acid alone.

**Table 4:** Micronutrient and inflammation status of study participants (*n* = 100).

Biomarker	% with Anemia or Evidence of Deficiency
**Iron ^1^**	
Hb < 12g/dL	37
Uadjusted serum ferritin < 15 μg/L	45
Adjusted serum ferritin < 15 μg/L	67
Unadjusted soluble transferrin receptor > 8.3 mg/L	11
Soluble transferrin receptor > 8.3 mg/L	7
Iron deficiency and anemia	35
**Zinc**	
Plasma zinc < 70 μg/dL ^2^	34
**Vitamin B12**	
Serum vitamin B12 < 150 pmol/L	23
Serum vitamin B12 < 221 pmol/L ^3^	60
Methylmalonic acid > 271 nmol/L	75
Holotranscobalamin < 35 pmol/L	46
Plasma homocysteine > 13 μmol/L	59
**Composite vitamin B12 indicator ^4^**	
Elevated levels (cB12 > 1.5)	3
Adequate levels (−0.5 < cB12 < 1.5)	34
Low levels (−1.5 < cB12 < −0.5)	44
Possibly deficient (cB12 < −2.5)	19
**Folate**	
RBC folate < 748 nmol/L	70
**Iodine**	
Serum thyroglobulin, μg/L ^5^	13.4 (5.0, 21.3)
Urinary iodine < 100 μg/L	0
I/Cr ratio, μg/g ^6^	409 ± 415
**Inflammation**	
CRP ≥ 5 mg/L	22
AGP ≥ 1 g/L	23

Hb, hemoglobin; sTfR, soluble transferrin receptor; CRP, C-reactive protein; AGP, α1-acid glycoprotein. ^1^ Serum ferritin values were adjusted for CRP and AGP using the BRINDA regression equations; sTfR values were adjusted for AGP using BRINDA regression equations (26,27). ^2^ There was no adjustment for inflammation because there was no relation between plasma zinc and CRP or AGP (47). ^3^ Vitamin B12 deficiency and insufficiency. ^4^ cB12 = log_10_[(holoTC × B_12_)/(MMA × Hcy) ] − [3.79/(1 + [age/230]^2.6^)] (31). ^5^ There is currently no cut-off for serum thyrogobulin levels in WRA. The median (first, third quartiles) is presented. ^6^ Urinary iodine to creatinine ratio. Mean ± SD is presented.

## Data Availability

The complete dataset and study forms will be made available online at osf.io three years after the completion of data collection.

## Supplementary Materials

The following supporting information can be downloaded at: https://www.mdpi.com/article/10.3390/nu15133024/s1. Table S1: Micronutrient and inflammation biomarkers of study participants; Table S2: Energy and macronutrient intake of study participants.
