# Supplementary material for: Women in Selected Communities of Punjab, India Have a High Prevalence of Iron, Zinc, Vitamin B12, and Folate Deficiencies: Implications for a Multiply-Fortified Salt Intervention
Source: Nutrients. 2023 Jul 3;15(13):3024. doi: 10.3390/nu15133024 (PMC10346790; doi:10.3390/nu15133024)
Supplement: Supplementary file 1 [file nutrients-15-03024-s001.zip › nutrients-2449060-supplementary.pdf]

Supplementary Materials

Table S1: **Micronutrient and inflammation biomarkers of study participants**

Biomarker	$\bar{x}$ ± SD or median (IQR)
**Iron**	
Hb, g/dL	12.4 ± 1.5
Uadjusted serum ferritin, µg/L	16.7 (9.7, 33.2)
Adjusted serum ferritin, µg/L	10.2 (4.9, 19.5)
Unadjusted soluble transferrin receptor, mg/L	4.7 (3.7, 6.3)
Adjusted soluble transferrin receptor, mg/L	3.3 (4.2, 5.7)
**Zinc**	
Plasma zinc, µg/dL	72.8 (67.5, 80.2)
**Vitamin B12**	
Serum vitamin B12, pmol/L	191 (154, 256)
Methylmalonic acid, nmol/L	603 (269, 944)
Holotranscobalamin, pmol/L	38.9 (28.8, 63.0)
Plasma homocysteine , µmol/L	14.59 (11.16, 19.87)
**Folate**	
RBC folate, nmol/L	623 (448, 796)
**Iodine**	
Serum thyroglobulin, µg/L	13.4 (5.0, 21.3)
Urinary iodine, µg/L	198.1 (180.0, 221.7)
Urinary creatinine, mg/dL	73.1 (41.1, 113.3)
I/Cr ratio, µg/g	409 ± 415
**Inflammation**	
CRP, mg/L	1.6 (0.7, 4.8)
AGP, g/L	0.8 (0.7, 1.0)
Malaria RDT, positive	0 (0)

n = 100; The data are means ± standard deviation for normally distributed biomarkers. Median (IQR) presented for skewed biomarkers. Hb, hemoglobin; CRP, C-reactive protein; AGP, α1-acid glycoprotein. Serum ferritin values were adjusted for CRP and AGP using the BRINDA regression equations(25); soluble transferrin receptor values were adjusted for AGP using BRINDA regression equations(26)

Table S2: Energy and macronutrient intake of study participants

	Mean ± SE	Median (IQR)
Energy, kcal/d	1768 ± 44	1719 (1471, 2014)
Protein, g/d	48 ± 1	46 (38,56)
Carbohydrate, g/d	246 ± 6	204 (239, 281)
Fat, g/d	66 ± 2	64 (53,77)

n=100; The data are mean ± standard error; Energy and macronutrient intakes are adjusted for usual intakes using Simple Macro (39)
